# Care in the time of COVID: An interpretative phenomenological analysis of the impact of COVID-19 control measures on post-partum mothers’ experiences of pregnancy, birth and the health system

**DOI:** 10.3389/fpsyg.2022.986472

**Published:** 2022-09-23

**Authors:** Mikhayl A. von Rieben, Leanne Boyd, Jade Sheen

**Affiliations:** ^1^School of Psychology, Deakin University, Burwood, VIC, Australia; ^2^Monash University, Eastern Health, Melbourne, VIC, Australia; ^3^School of Psychology, Deakin University, Burwood, VIC, Australia

**Keywords:** COVID-19, pregnancy, post-partum, healthcare, family

## Abstract

**Background:**

Findings suggest pandemic control measures have modified maternal health practices, compromising the quality of care provided to new and expectant mothers and interfering with their birthing experiences. For this reason, this study explored the lived experiences of post-partum Victorian mothers during the pandemic as well as the potential influence of control measures over their perceptions regarding the health system.

**Methods:**

This study used a qualitative approach. Recruitment was conducted between May and June 2021, using both the Australian Breastfeeding Association’s social media pages and snowball recruitment. Interviews were semi-structured using open-ended questions relating to key themes. Seven Victorian post-partum mothers were identified and their transcripts analysed using Interpretative Phenomenological Analysis.

**Results:**

Mothers described how unexpected changes to maternal care exacerbated feelings of uncertainty regarding pregnancy and birth. Mothers also differentiated between impacts by the health system and the role healthcare professionals played in moderating these effects. Whilst visitor restrictions provided some benefit, restrictions to familial and social support left many of the mothers feeling alone during their pregnancy and interfered with their immediate post-partum experience.

**Conclusion:**

This study illustrates the importance of evidence-based practice in maternal care and provides insights for both health professionals and policy analysts in developing new or modifying existing guidelines that better balance the needs of expectant and post-partum mothers with pandemic control measures.

## Introduction

On the 11th of March 2020, the World Health Organisation (WHO) declared Covid-19 to be a global pandemic, prompting governments across the world to implement a range of strategies to control viral transmission (Hale et al., 2021). In the Australian context, these control measures included isolation or quarantine of both infected and potentially infected persons, contact tracing, mandatory use of face masks, border closures, cessation of international travel, social-distancing and community lockdowns (Australian Government Department of Health, 2020; AGDH). Whilst these measures may assist in reducing transmission (Ayouni et al., 2021), emerging research suggests the pace and scale at which these strategies have been implemented have compromised health practices, placing service-users at risk (Semaan et al., 2020; Woolliscroft, 2020; Hugelius et al., 2021; Kendzerska et al., 2021; Lalor et al., 2021).

Recent evidence also suggests pre- and post-partum mothers may be particularly affected by such changes to care given the additional vulnerability associated with pregnancy and birth (Riley et al., 2021; Vazquez-Vazquez et al., 2021; Wilson et al., 2021). For example, prevalence studies comparing levels of anxiety (25–64%) and depressive symptoms (26–37%) amongst new and expectant mothers during the pandemic have shown significant increases compared to earlier samples (10 and 15%; Ceulemans et al., 2020; Lebel et al., 2020; Liu et al., 2020; Molgora and Accordini, 2020). Whilst risk of infection was cited as a major source of distress during this time (Molgora and Accordini, 2020; Ravaldi et al., 2020; Naurin et al., 2021), qualitative analyses have also revealed how control measures impacted women’s perceptions regarding their received care and influenced their pre- and peri-partum experiences, resulting in feelings of isolation, guilt-tampered happiness, uncertainty regarding pregnancy and loss of control over the birthing process (Chivers et al., 2020; Coxon et al., 2020; Ravaldi et al., 2020; Riley et al., 2021; Wilson et al., 2021). These findings are concerning as women’s psychological health during pregnancy and childbirth are major predictors of post-partum well-being (Choi et al., 2017; MacKinnon et al., 2017; Molgora et al., 2020).

Recent reviews suggest such changes in care reflect an increasing pressure on healthcare professionals (HCPs) to balance care with control measures, with preparations for Covid taking precedent over evidence-based practice (Renfrew et al., 2020; Kotlar et al., 2021; Lalor et al., 2021). These reviews are supported by research involving 714 maternal and newborn HCPs which found widespread concerns regarding the ways control measures have modified and potentially reduced the quality of care provided to peri- and post-partum mothers (Semaan et al., 2020). Such modifications include banning support partners; separating newborns from Covid-19 positive mothers; use of masks and face shields for HCPs, mothers and support partners; Covid screening prior to in-patient visits; limiting access to in-person support services; transitioning to telehealth; and, reducing outpatient visits or length of stay post-birth (Semaan et al., 2020; Boelig et al., 2020a,b). Additionally, despite women increasingly requesting home-births due to concerns regarding infection, HCPs have become less likely to support the practice due to social-distancing guidelines (Coxon et al., 2020).

These modifications are of serious concern as restricting women’s access to professional and familial support may increase the risk of unplanned or unsupervised home-births or reduce uptake of post-natal care, increasing the risk of maternal and infant death (Zielinski et al., 2015; Roberton et al., 2020). Previous studies have also shown significant associations between pre- and post-partum women’s psychological wellbeing and the quality of their spousal- and social-support networks (Biaggi et al., 2016; Ilska and Przybyła-Basista, 2017; Tani and Castagna, 2017; Figueiredo et al., 2018; Friedman et al., 2020). Thus, reducing access to spousal, familial and social support may exacerbate feelings of isolation and maternal distress, especially for those with high-risk pregnancies (Riley et al., 2021; Wilson et al., 2021). Furthermore, control measures such as social-distancing and masks may interfere with mother-infant bonding (Tscherning et al., 2020) or reduce the likelihood of establishing consistent breastfeeding practices (Vazquez-Vazquez et al., 2021).

Whilst there is an emerging body of evidence pointing to the impact of control measures on new and expectant mothers, few studies to date have explored the lived experiences of Australian mothers regarding the pre and post-partum period during the pandemic (Wilson et al., 2021). Furthermore, differences between and within countries regarding the severity, frequency and duration in the use of control measures (McKenzie and Adams, 2020) may result in different experiences of the health system (HS) and, subsequently, the pre and post-partum period. As such, more research is needed to understand the lived experiences of pre and post-partum mothers during this time. For these reasons, this study was guided by the following research questions:

1. What is it like for pre- and post-partum mothers to experience daily living during the coronavirus pandemic?
2. What influence, if any, have control measures had on post-partum mothers’ perceptions regarding the HS?

## Materials and methods

### Methodology

This study employed a qualitative, inductive and constructivist approach to research design, drawing insights from participants’ subjective experiences. This means the researcher was viewed, not as a neutral observer but as an inextricable component of the meaning-making process (Creswell, 1998; Smith and Osborn, 2015). To compensate for this, Interpretative Phenomenological Analysis (IPA) was used to develop a collaborative and highly detailed idiographic examination of the participants’ experiences (Smith and Osborn, 2015). As such, any meaningful insights presented here should be considered a ‘co-construction’ between both the participants and the researcher (Osborn and Smith, 1998; Smith et al., 2009). However, the double-hermeneutic of IPA also means such insights may still be influenced by the researcher’s own biases (Tuffour, 2017). To minimise the impact of these, the primary researcher maintained a running commentary of their impressions, recorded within the margins of the text during analysis (Smith et al., 2009) and engaged in continuous reflexive dialogue with the principal investigator throughout the study (Austin and Sutton, 2014). Reflexivity refers to the means by which a researcher actively considers their role in the research process, including any prior assumptions and experiences, and how this role may shape the data they collect or the insights they produce (Dodgson, 2019). A reflexive statement for this study is provided in Appendix A.

### Recruitment

Ethics approval for the study was provided by Deakin University as part of a larger study (see Appendix B). Recruitment for this study was conducted between May and June 2021 by sharing flyers *via* the Australian Breastfeeding Association (ABA) social media pages, as well as various Australian new-mother Facebook support groups. Both fathers and mothers were eligible to participate if they were expecting or had an infant under the age of 12 months. Prospective participants were sent a plain language statement (PLS), consent form and demographics questionnaire and were offered a $50 gift voucher as compensation for their time due to the length of interviews. Of 48 respondents, only 22 parents signed and returned the consent form, leaving a response rate of 46%. This low response rate is in-line with other qualitative and mixed methods studies focusing on mothers’ experiences of post-natal care and may be associated with the additional obligations parents experience post-birth (Malouf et al., 2019). Following this initial sample, four participants could not be reached in order to schedule an interview and one cancelled due to a death in the family, leaving a total of 17 participants.

### Participant selection

As a primary focus of this study included pre- and post-partum mothers’ experiences of the HS, participants were required to have English as their first language in order to control for issues associated with language barriers (Al Shamsi et al., 2020). Furthermore, only participants who resided in Victoria were included due to the variability in restrictions across different states (Stobart and Duckett, 2021). Of the remaining participants, three were identified as being in the pre-partum period with two of these being married to one another and one participant identifying as male. Whilst the study included both fathers and mothers, it was thought the inclusion of a single male would be insufficient to conduct a gendered-lens analysis. Similarly, the inclusion of only two pre-partum parents could not be justified as their experiences were thought to be too distinct from that of post-partum parents, thus reducing participant homogeneity required for IPA (Smith et al., 2009). Whilst Smith et al. (2009) advise using a sample size of three, others suggest 6–10 participants may be needed to examine convergence and divergence within themes (Morse, 2000; Marshall et al., 2013).

### Participants

The final sample consisted of seven women aged 31–43 years. All participants identified as White/European Australian, married, tertiary educated and residing in Victoria. Further characteristics of the sample are presented in Table 1.

**Table 1:** Participant profile.

Pseudonym	Age	Care	Relational status	# of children
Annie	35	Public	Married	3
Beatrice	34	Private	Married	1
Cordelia	31	Public	Married	3
Delilah	43	Private/Public	Married	3
Felicia	38	Private	Married	2
Georgia	31	Public	Married	1
Irene	34	Public	Married	1

### Data collection

Interviews took place *via* Zoom, which is a free and readily-accessible virtual meeting software that can be used *via* laptop or mobile phone (Lobe et al., 2020). Participants were asked to confirm whether they had received the PLS and that they consented for the interview being recorded. After this, the interview began with questions following a semi-structured approach. Whilst IPA does not adhere to a strict interview guide (Smith et al., 2009), prompts and open-ended questions following key themes were employed to explore participants’ lived experiences (see Appendix C for a complete list of interview questions). The duration of the interviews ranged between 45 and 90 min and were transcribed verbatim. Transcripts were then sent to participants to confirm their validity (Goldblatt et al., 2011). All participants approved their transcript for de-identification and analysis with only three participants making minor revisions in order to clarify their relationship to external family members or to further de-identify aspects of their work.

### Analysis

**Figure 1:**
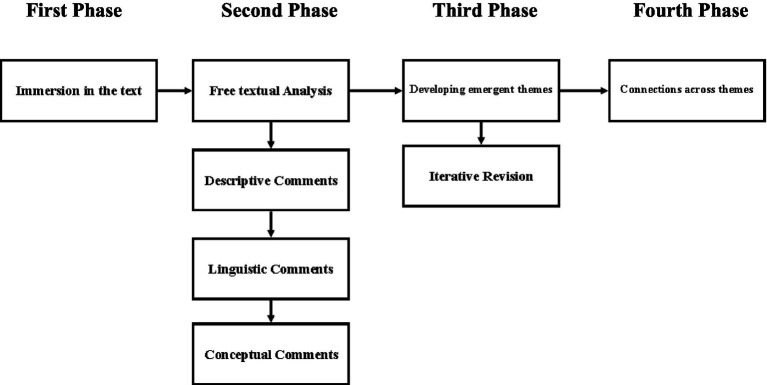
Overview of the analytic process.

Analysis of the data followed the method outlined by Smith et al. (2009). An overview of the analytical steps taken is provided in Figure 1. The process for analysis began with the first author immersing themselves in the data by reading transcripts, line-by-line, multiple times whilst listening to the interview recordings. The second phase involved the first author engaging in a free textual analysis approach, whereby the researcher’s initial thoughts and reactions to the text were recorded. Annotations of the text were then identified based on the nature of their content (e.g., descriptive, linguistic or conceptual). It’s important to note that, compared to descriptive or linguistic coding, conceptual coding involves a more interpretative and interrogative approach to the text in an attempt to move beyond a surface level understanding of the data (Smith et al., 2009). The purpose of this approach was to develop succinct statements that captured the essence of the psychological phenomena whilst retaining something of the particular, resulting in comments that were both grounded and conceptual in nature.

The third phase involved identifying subthemes between codes and grouping them together, with such collections constituting emergent patterns of meaning regarding the text. These subthemes were then discussed with the principal investigator and iteratively revised in order to ensure they were grounded in the data. This involved testing assumptions and biases regarding the findings, comparing themes to existing literature and contrasting annotations to the original text. The final phase, involved grouping these subthemes into superordinate themes. Criteria for the selection of superordinate themes were based on their presence and persistence amongst all or a majority of the seven participants as well as their coherence and logical fit with existing research.

## Credibility of the findings

Whilst validity is important for qualitative research, the criteria for determining validity is debated (Smith et al., 2009). To improve credibility of the findings this study observed the criteria set out by Yardley (2000) which focuses on (1) sensitivity to context, (2) commitment and rigour, (3) transparency and coherence, and (4) impact and importance. Contextual sensitivity was of particular importance for this study given the subject of investigation and influenced all stages of the research, from selection of the research design and methods to the collection and analysis of data. Similarly, commitment and rigour were exercised through strict adherence to the guidelines established by Smith et al. (2009).

## Results

Analysis led to the identification of three superordinate themes: (1) The ‘Control’ in Control Measure, (2) Care in the Time of Covid, and (3) Baby Space. An overview of the superordinate themes and their respective subthemes are presented in Table 2 with additional exemplificative quotations provided in Appendix D.

**Table 2:** Superordinate themes and their subthemes.

Superordinate themes	Subordinate themes
The ‘Control’ in Control Measure	Changes in Protocols
	Regaining Control
Care in the Time of Covid	Compared to Covid
	At Arm’s Length
	Masks and Midwives
	Under Pressure
Baby Space	It Takes A Village
	Together Alone
	Our Little Bubble

### Theme 1: The ‘control’ in control measure

The first theme related to the ways in which changes to protocols exacerbated mothers’ feelings of uncertainty regarding their pregnancy, prompting them to employ their own strategies for reducing anxiety. This theme emerged through two subordinate themes: (1) Changes in Protocols; and (2) Regaining Control.

#### Changes in protocols

For all of the mothers, the general uncertainties surrounding pregnancy and birth were amplified by the frequent and unexpected changes to hospital protocols associated with the Covid restrictions, as Cordelia explained “*my anxiety was a lot higher because things were ever-changing.*” Whilst these changes covered a range of aspects regarding their pregnancy and peri-partum experience, there were uniting themes between mothers. For example, Georgia recalled how:

*…advice kept changing, even between different hospital staff, as to what would be allowed at the time. Whether I would need a Covid test, whether I would have two people at the birth …or just my partner. Whether I would be allowed to have certain pain relief options…* (Georgia)

Changes to policies regarding partner participation were particularly problematic, leaving some of the mothers unsure as to whether their partners would be allowed to stay and support them during recovery. “*…It was a big worry if he might be kicked out or not allowed to be there*” (Felicia). The uncertainty surrounding visitors also made it difficult for mothers to plan their stay. “*I had to pack a lot of clothes for me and the baby*” Georgia explained, “*…you could not give your Mum some dirty washing and ask her to bring it back…*” Beatrice related similar feelings of uncertainty regarding her maternal child health visits post-discharge: “*…It was unknown whether they could make a face-to-face visit or whether it would be via Zoom, which was very anxiety provoking because you are a new mum.*”

#### Regaining control

The mothers often described feelings of anxiety and loss of control over their pregnancy or birth, however, the ways in which the mothers responded to this uncertainty varied considerably. Georgia for instance “*…tried really hard to be prepared …by trying to check up on …the hospital restrictions …how they were changing week on week.*” Georgia also credited the use of midwife engagement programs and “*…anti-natal classes in whatever form they were*” as well as the support of her partner. Irene prepared herself by researching as much as she could regarding her caesarean and developed a birthing plan, however, feeling unheard, Irene became frustrated and demanded the level of care she needed:

*…I said, I want skin-to-skin as soon as possible, I want to try and breastfeed. And she goes, “oh we’ll see what we can do” …and I said “no …that’s what I want. I need to be able to do that”.* (Irene)

This contrasted with Cordelia’s birthing experience, the success of which she attributed to the support of her midwife who “*…made the whole process less stressful …she was very calming*.” For both Beatrice and Delilah, the ability to combine private and public care provided them with an additional level of control. “*I went through the private system*” Beatrice explained, “*…so I was able to see an obstetrician and keep all those normal appointments.*”

### Theme 2: Care in the time of Covid

The second superordinate theme revealed the scale of the impact control measures had on the care mothers received and the role HCPs played in mitigating or exacerbating these effects. This theme was reflected in four superordinate themes: (1) Compared to Covid; (2) At Arm’s Length; (3) Masks and Midwives; and, (4) Under Pressure.

#### Compared to Covid

Whilst all of the mothers recognised the exceptional circumstances surrounding their pregnancy some of the mothers expressed feeling as though their needs were considered secondary compared to Covid, as Georgia recalled “*…sometimes it felt like pregnant women …were not being prioritized …from the beginning it felt like the preparations for Covid meant that support opportunities were denied.*” These views aligned with Irene’s experience, where preparations for Covid had prevented her from having a water-birth. “*…they had actually just started stockpiling furniture in the birthing suites with the hot tubs …So – because of Covid …they needed more room for extra beds*” (Irene).

This focus on Covid led many of the mothers to question the care they were receiving in a way they had not before, however, the mothers also made distinctions between the care provided to them by various HCPs and the HS itself. “*…I did not feel [the medical supports] were ideal, not for any medical professional’s doing but because of the lockdown situation, I did not feel as supported as I could have during pregnancy*” (Georgia). Both Delilah and Beatrice made a further distinction between private and public care:

*From the health care providers that I have proactively sought …I felt like those two examples went out of their way to make me feel supported. From the public side of things …I have not felt supported at all.* (Beatrice)

#### At arm’s length

A significant and systemic change in the way care was provided during the pandemic involved the transition from in-person care to telehealth. Whilst all of the mothers viewed telehealth as a poor substitute for in-person care, there were some differences in the way it impacted them. For example, Delilah’s concerns regarding miscarriage meant she was particularly worried about the risk of infection during pregnancy and wanted to reduce any hospital visits. “*…you do not want to go to a doctor’s surgery …if you could avoid it, you do not want to be anywhere like that*” (Delilah). Instead, Delilah recalled how her private obstetrician made her feel empowered by letting her choose whether to use “*…telehealth if you wanted to or if you wanted to go in.*” This contrasted with Annie’s experience of public care who described how she needed to “*…fight to get seen and not do Telehealth.*” Cordelia was also skeptical about the level of care telehealth could provide:

*There’s things that you can’t really …assess via a phone or a computer and it’s a lot less personal, and particularly, things like breastfeeding is quite an emotional thing for a lot of people so I think face to face support provides a lot more for that.* (Cordelia)

This view aligned with Georgia’s experience regarding breastfeeding, which she described as being “*…more of a physical training experience*,” suggesting there were aspects to breastfeeding that could not be conveyed *via* video. Anti-natal classes delivered *via* teleconference were similarly problematic. “*It was more difficult to ask questions just because it’s a very awkward format and …they were not as long as they would have been if they were in person*” (Georgia). Felicia also noted that building rapport was more difficult through telehealth, resulting in her being less likely to disclose sensitive information, “*…[you are] not necessarily as honest or you know forthcoming when you feel like you are on a phone call and someone’s watching the clock*.” With regards to post-partum care, Beatrice added “*…even just logging into a Zoom meeting and getting the camera sorted and being able to hold a conversation with the camera whilst holding a baby is really difficult*.”

#### Masks and midwives

Masks also played a salient role in creating a barrier between the mothers and their respective HCPs. For example, despite having built a rapport throughout the course of her pregnancy, Beatrice recalled how she had only ever seen her obstetrician once without a mask. “*…I saw his whole face which was quite kind of shocking …he’s been treating me for 9 or 10 months and I’ve never really seen his face*.” During the birth itself, Beatrice described how:

*All of the nursing staff and doctors wore masks the entire time even in the …birthing ward. So that made it difficult just to communicate or even see a nurse’s expression which those nonverbal cues are really important to establishing effective communication.* (Beatrice)

Three weeks before she was due, Georgia developed pre-eclampsia and was admitted to hospital. She recalled how she had “*…finished work on the Thursday night and then was induced on the Friday, which was intense*.” Georgia also described how the sudden transition from work to giving birth, combined with the hospital staff wearing masks, left her feeling dissociated from her birthing experience. “*I wasn’t wearing a mask but I was the only one beside from the baby not wearing a mask and that was a really weird de-humanizing experience …it felt really strange*” (Georgia).

#### Under pressure

At times the mothers described how various HCPs would mitigate or exacerbate the impacts of the HS. For example, Georgia noted that HCPs around her appeared to be under a considerable amount of pressure. “*…sometimes it felt like everyone was very, very rushed and I do not know how that might have affected some of the support I’ve got in hospital*” (Georgia). This pressure extended to Annie who was discharged 12 h after giving birth. “*I think I wasn’t ready to be discharged. I had the worst physical recovery of any of my pregnancies, like it took me like six weeks …I was not well*” (Annie).

These experiences pointed to the role of the HCP as an important factor in assisting mothers in navigating the HS during various levels of restrictions, however, other factors such as the seniority of the HCP or their fear of exposure appeared to moderate their capacity to do this. For example, Felicia described how “*the nurse I had …was pretty anxious about seeing the community so it was definitely a stressful experience with the baby crying and getting measured…*” In another example, Annie recalled how she was contacted and asked to return for an emergency ultrasound soon after being discharged. When she arrived with baby the reception asked her to leave due to restrictions on children. After contesting the policy Annie left the hospital, however, “*…10 min later they were super apologetic, took the baby for me, loved the baby, gave me the scan*.” Annie suggests this change in approach was due to senior staff being more flexible regarding hospital guidelines. “*…someone else had overhead the situation, someone more senior …who was able to look at it just with some common sense*” (Annie).

### Theme 3: Baby space

The third superordinate theme captured how restrictions to services (subtheme 1) and support partners (subtheme 2) left mothers feeling alone during pregnancy and interfered with those brief moments following birth, taking mothers away from being present with baby (symbolically represented here as ‘baby space’; subtheme 3) and disrupting the rituals and meaning-making processes associated with baby’s arrival.

#### It takes a village

Many of the mothers described how additional services such as “*…birthing classes, breastfeeding support …pelvic floor clinic …those sorts of things*” (Cordelia) were scaled back or stopped completely, with others migrating to virtual spaces or requiring participants to socially distance. “*…we did a group kind of physio class and …they would come to you to stop …people circulating*,” Felicia recalled. However, these restrictions meant “*…the ability to meet other expectant parents was non-existent*.” (Beatrice), thus denying mothers the opportunity to develop support networks. This absence of in-person support particularly affected first-time mothers like Georgia, who struggled to adjust to everyday challenges such as breastfeeding. “*…my parent’s group started quite a bit later than it would have if not for Covid so I was pretty desperate at that point for some sort of in-person support network…*” (Georgia).

#### Together alone

For all of the mothers, the loss of community increased their dependency on their partners, resulting in the parent-dyad becoming stronger. “*…because we have had no support at all…*” Irene explained, “*…we have realised that we are a very good team unit, and I knew that always, but it’s really solidified that*.” However, this effect was tempered by the restrictions placed on support partners resulting in mothers feeling alone in their birthing journey. “*In normal times, I could have had him there …all day, just had a chat, hand him the baby …it was very isolating*” (Irene).

For first-time mothers, or those who had experienced high-risk pregnancies, being alone also compounded their feelings of uncertainty. Delilah recalled how “*…we’d had experiences of miscarriage, quite a few and there was quite a bit of anxiety about going for scans and not having [husband] there was a bit shit*.” Some of the mother’s also described their partners’ disappointment at being unable to attend their baby’s ultrasound; “*He was like ‘that’s not fair’ …you know both for support for me and also for him to experience*” (Delilah). For Annie, the absence of her partner from those early appointments resulted in a delay in him mentally preparing for baby’s arrival. “*It took him longer to catch on that we were having a baby*,” Annie explained. “*Obviously he knew but …I feel like he had trouble engaging in those conversations earlier …and I wonder whether because it wasn’t as real for him …as it was for me*” (Annie). Whilst private care appeared to offer some flexibility, the ability for partners to attend appointments was largely dependent on the timing of restrictions.

*I was very lucky I went through the private system, so I was able to …keep all those normal appointments and actually [husband] was able to attend I think all bar one of them due to the varying levels of lockdown.* (Beatrice)

In some cases, mothers were able to mitigate the impact of this restriction through the use of telehealth. “*…I’d have [husband] on speakerphone …he could hear what the midwife was saying, he could hear the heartbeat, he could hear how it was growing okay, and then what the next appointment would entail*” (Irene). However, this method also had its limits. For example, at 36 weeks Irene was informed that her baby had stopped growing: “*…it was the only one [husband] did not call in on, because all my appointments were fine, absolutely healthy…*.” Instead, Irene lamented the fact that her partner would not find out about baby’s complications until she arrived home. “*…that would have been nice to have him there when I heard that, because I had to wait to get home …instead of him hearing firsthand*” (Irene).

#### Our little bubble

Despite their experiences, most of the mothers described how the restrictions on visitors provided them with a rare opportunity to “*…just be safe in our own little bubble*” (Beatrice). For example, Felicia described how “*…the hospital was a lot calmer, like nobody else was having visitors, there was less people on the floor, less noise. I think the staff were more relaxed because they were just dealing with patients…*”. Felicia also described how visitor restrictions had provided her with “*…permission not to see people …just to focus on you and your new baby, and the immediate people in your house*.” However, “*…after it got beyond …the first few weeks*,” Cordelia explained, “*…I found it really difficult and I felt very angry…*.”

These experiences pointed to a brief and intimate window during the first few weeks of birth, where the mothers required both the closeness and support of their immediate family as well as respite from their wider social obligations. However, having to demand care forced them out of this bubble, thus making “*…it harder to focus on the baby, because it takes you into another space other than that baby space*” (Annie). For Cordelia, the interference with and sometimes outright denial of these moments by the various control measures, resulted in a profound sense of loss:

*…that was, I guess, taken from me and my daughters …it was just …grieving not being able to have my daughters involved in the pregnancy, grieving family and friends not meeting him until he was two months old …grieving my expectations …versus what actually transpired.* (Cordelia)

## Discussion

In this study, seven Victorian mothers’ pre- and post-partum experiences of the HS were examined in-depth using IPA (Smith et al., 2009). The mothers’ accounts revealed how control measures significantly modified the care they received which in turn influenced their experiences of the pre- and post-partum period. Such changes included transitioning from in-person support to telehealth, the use of face masks by HCPs, banning support partners during appointments, restrictions on visitors, reduced length-of-stay, reduced birthing options and the scaling back or cessation of ante- and post-natal services. These findings align with previous studies, which identified concerns by mothers (Ravaldi et al., 2020; Riley et al., 2021; Wilson et al., 2021) and maternal HCPs (Semaan et al., 2020) regarding changes to maternal health practices compromising care.

The mothers also described how the uncertainty surrounding these changes reduced their sense of control over the birthing process. As a result the mothers employed various strategies to restore their personal agency, such as monitoring changes to restrictions, electing private over public care, demanding their preferred level of care, developing a birthing plan or seeking out additional support services. Previous studies have highlighted the importance of maternal decision-making during pregnancy and birth (Cook and Loomis, 2012; Yuill et al., 2020), however, it should be noted the mothers in our study were tertiary educated which may influence their ability to implement other strategies or to access and navigate private care. As such, unexpected changes to protocols may have an even greater impact for at-risk mothers. To compensate for this, HCPs should consider adopting a participatory approach that describes the relative risks to mothers and engages them in the decision-making process (Vahdat et al., 2014; Reingold et al., 2020).

The mothers also described how HCPs helped to moderate their experiences of the HS pointing to seniority, fear of infection, hospital pressure as well as differences between private and public care as important factors. Preparations for Covid were also identified as impacting mothers’ experiences by reducing their birthing options and leaving them feeling as though their needs were less important compared to Covid. Renfrew et al. (2020) suggests such changes are a result of the pressure midwives face, working within an increasingly uncertain environment. For example, the risk of staff exposure means HCPs have to self-isolate, putting additional strain on services already stretched thin (Renfrew et al., 2020). In response to this, midwives may revert to a ‘command-and-control’ approach as opposed to midwifery-led, women-centred and context-dependent care (Renfrew et al., 2020). Additionally, both students (Renfrew et al., 2020) and HCPs (Søreide et al., 2020) may learn to prioritise preparations for Covid over care, furthering influencing future practice.

Whilst telehealth has been promoted as a means of improving care by way of reducing patient and staff exposure (Diamond et al., 2020; Fryer et al., 2020), concerns have been raised regarding the ways in which telehealth dehumanises clients and limits personalised care (Neville, 2018). This was reflected in the mothers’ experiences, which described telehealth as being limited, impersonal and less comprehensive. First-time mothers who had difficulty establishing breastfeeding practices further illustrated the importance of in-person support. Being physically present is thought to be an essential component to care (Oudshoorn, 2009) and forms the basis of other important midwifery practices such as effective communication, advocacy, presence and partnership (Bradfield et al., 2019). Whilst maternal health practices may need to adjust to the challenges set by the pandemic, the use of telehealth may not be appropriate for all mothers. Instead, the decision to use telehealth should be based on the needs of service-users, with mothers being empowered to elect in-person services where needed. Healthcare leaders may also empower midwives by promoting evidence-based practices such as midwifery-led care (Ricchi et al., 2019).

Restrictions on women’s access to spousal, familial and social support also figured strongly in our study, with mothers reporting feelings of isolation and loneliness in their birthing journey. Experiences of loneliness due to restrictions have also been reported by peri-partum mothers in the United States of America (Farewell et al., 2020) and United Kingdom (UK; Riley et al., 2021). According to Diamond et al. (2020), such restrictions reflect a prioritisation by health leaders for the biomedical above, or even at the exclusion of, the psychological and social aspects of the biopsychosocial model. In this way, families and partners are seen as superfluous to women’s health, rather than an important source of support. This is unfortunate, as previous studies have consistently shown significant associations between pre- and post-partum women’s psychological well-being and the quality of their familial- and social-support networks (Biaggi et al., 2016; Ilska and Przybyła-Basista, 2017; Tani and Castagna, 2017; Figueiredo et al., 2018; Friedman et al., 2020). Furthermore, failing to include families denies members the opportunity to participate in the kinds of rituals and meaning-making processes associated with baby’s arrival (Imber-Black, 2002), potentially impairing family functioning and reducing quality of life for its members (Fiese et al., 2002).

Whilst Diamond et al. (2020) recommend the use of telehealth to address mothers’ social needs, the accounts in our study revealed mothers who were at significant risk of miscarriage still felt alone in their pregnancy despite the use of these services. This suggests that mothers may prefer differing levels of support subject to their needs. Excluding fathers from appointments also raises serious ethical concerns regarding the ways in which the HS prioritises the needs of mothers and fathers differently, particularly in regards to the non-disclosure of foetal complications or miscarriage. Previous research suggests fathers have different needs to mothers during miscarriage, abnormal birth or stillbirth, including the need for information and to be a part of the decision-making process, and may become frustrated if they are unable to support their partners during this time (Ellis et al., 2016). Alternatively, inclusion of fathers during pregnancy offers additional benefits including promoting breastfeeding and establishing a supportive environment for mothers post-partum (Kothari et al., 2019). Given the improved availability and uptake of vaccines as well as the attenuated severity of the virus (AGDH, 2022), it’s recommended the AGDH adopt similar strategies to the April revision of the UK’s National Health Service (2021) guidelines. Additionally, HS may reintroduce policies that support women’s access to families and support partners throughout the birthing journey and promote paternal participation in the birthing process.

Lastly, the accounts of the mothers in our study suggest that mask-wearing by HCPs also acted as a barrier for effective care by reducing the mothers’ ability to communicate or build rapport with staff. Whilst few studies to date have explored mothers’ experiences in relation to the use of masks by HCPs (Gutschow and Davis-Floyd, 2021), facial expressions have been shown to be important for communicating empathy between patients and practitioners (Holland et al., 2021) with masks reducing patients’ perceptions of empathy (Wong et al., 2013). In many ways, HCPs represent the human face of the HS. Thus, when all interactions with HCPs are conducted behind face masks, the human element of health care may be negated, instead transforming staff into faceless functionaries of the HS (Rushton and Edvardsson, 2021).

The individual account of dissociation during labour by one of the mothers and its association with HCPs wearing masks also appears to be particularly novel. Whilst predisposing factors such as childhood psychopathology should be considered, precipitating factors such as one’s perception of care and negative appraisals of labour have been associated with the onset of peri-traumatic dissociation (PD; Choi and Seng, 2016). Alternatively, being able to observe empathy in HCPs has been shown to reduce perinatal trauma, thus acting as a buffer against PD (Krausé et al., 2020). A tentative hypothesis then might be that the inability to observe HCPs emotional expressions during labour may contribute to negative appraisals of labour, potentially acting as a precipitating factor for PD. Whilst this finding should be considered with caution, the impact of mask wearing by HCPs on mothers’ perinatal experiences warrants further investigation as PD is a significant predictor for PTSD, depression and impaired bonding following childbirth (Choi and Seng, 2016). HCPs may also benefit from screening mothers for predisposing factors using the Peri-traumatic Dissociative Experiences Questionnaire (Birmes et al., 2005), in order to determine whether masks should be avoided during labour.

### Study limitations and future research

Whilst qualitative studies are not generalisable by design, several limitations are acknowledged. First, the mothers in our study delivered their babies at different times and under varying levels of restrictions which may have impacted their pre and peri-partum experiences. Second, participant demographics reflected a White, educated, working-to-middle class cohort and therefore may exclude issues related to at-risk or minority groups. Another limitation of the study was that findings were not discussed with participants or HCPs other than those involved in the research. Future research should prioritise at-risk cohorts, including non-English speaking immigrants, who may not have access to traditional support networks or may be impacted by restrictions on international travel. Longitudinal data may also be useful in understanding the impact of reverting to earlier models of practice on mother and infant well-being. Further research may also be needed to understand the role of masks in relation to PD.

### Conclusion

The Australian government’s response to the COVID-19 pandemic has deeply impacted maternal health practices in Australia and left an indelible mark on the lives of the women at its centre. In our study, unexpected changes to care resulted in feelings of uncertainty which prompted mothers to supplement their care and restore a sense of agency over the birthing process. Whilst mothers indicated that restrictions to visitors offered some benefit, in many instances, restrictions to services and support networks left mothers feeling alone and unable to share in the meaning-making processes associated with baby’s arrival. Despite the utility in telehealth and masks for reducing staff and patient exposure, the mothers’ experiences in our study also suggested these measures restricted HCPs ability to communicate or be present, limiting their ability to deliver effective care. The apparent regression from evidence-based frameworks, such as the biopsychosocial model, towards earlier models of care is particularly concerning, as it is not clear what long-term implications these changes will have on maternal well-being. HCPs are integral to restoring balance, however, HCPs must be empowered to independently determine the correct balance between maternal care and preparations for Covid. We recommend HCPs re-introduce evidence-based practices such as permitting and promoting access to support partners and family during health visits as well as the peri-natal period. This may also be done in complimentary fashion with telehealth services in order to maximize women’s options and restore a sense of control over the birthing process. Overall, the findings in our study offer health professionals and policy analysts an opportunity to stop and reflect on the changes being made to maternal care and to ensure the holistic needs of both women and their families are considered when developing new or modifying existing guidelines for either current or future health crises.

## Data availability statement

The raw data supporting the conclusions of this article will be made available by the authors, without undue reservation.

## Ethics statement

The studies involving human participants were reviewed and approved by Deakin University Human Research Ethics Committee (HEAG-H 70_2020). The patients/participants provided their written informed consent to participate in this study.

## Author contributions

JS: supervision, conceptualisation, methodology, and writing (reviewing and editing), funding acquisition. MR: conceptualisation, investigation, data curation, formal analysis and writing – original draft preparation. LB: conceptualisation, methodology and writing (reviewing and editing). All authors contributed to the article and approved the submitted version. All authors contributed to the article and approved the submitted version.

## Funding

Funding for the project was provided by Deakin University as part of the first author’s honours thesis.

## Conflict of interest

The authors declare that the research was conducted in the absence of any commercial or financial relationships. That could be construed as a potential conflict of interest.

## Publisher’s note

All claims expressed in this article are solely those of the authors and do not necessarily represent those of their affiliated organizations, or those of the publisher, the editors and the reviewers. Any product that may be evaluated in this article, or claim that may be made by its manufacturer, is not guaranteed or endorsed by the publisher.
