# Supplementary material for: Care in the time of COVID: An interpretative phenomenological analysis of the impact of COVID-19 control measures on post-partum mothers’ experiences of pregnancy, birth and the health system
Source: Front Psychol. 2022 Sep 23;13:986472. doi: 10.3389/fpsyg.2022.986472 (PMC9537098; doi:10.3389/fpsyg.2022.986472)
Supplement: Supplementary file 1 [file Data_Sheet_1.docx]

Supplementary Material

# Appendix A: Reflexive Statement

## Reflexivity

Reflexivity refers to the means by which a researcher actively considers their role in the research process, including any prior assumptions and experiences, and how this role may shape the data they collect or the insights they produce (Dodgson, 2019).

## Prior assumptions and experience

Within the context of the current study, the primary researcher (MVR) acknowledges that his position as a male, a student and being from a non-clinical background, may have shaped both his and the participants’ perceptions regarding the content and nature of their interactions or influenced the researcher’s analysis of the text. This in turn may have influenced participants’ willingness to discuss their personal experiences, the type of information disclosed to the researcher, as well as the researcher’s interpretation of the experiences discussed. For example, whilst all participants appeared open and collaborative MVR recognises that female participants may not have felt comfortable disclosing personal information regarding breastfeeding practices to men or may have previous experiences of domestic violence. Furthermore, as a student with no clinical experience MVR recognises that the significance of emergent themes may have been weighted disproportionately towards personally relevant or recognisable issues, excluding those outside the researcher’s understanding. However, MVR believes that his personal experience of having supported his partner during their child’s birth under various restrictions may have also helped to align the researcher and the participants, offering a more robust and accurate interpretation of the data.

## Awareness of social setting and the social ‘distance’ between the researcher and the researched

All of the interviews were conducted via Zoom as the researcher resided in South Australia and the participants resided in various parts of Victoria. Whilst participants reported finding the medium convenient for balancing infant care with the interview, the researcher was aware that some participants may have varying levels of computer literacy, or experience various interruptions and technical difficulties during the course of the interview. For example, the researcher was conscious of delays in audio-video transmission and the impact of ‘talking over’ one another. As such, the researcher used facial gestures as minimal encouragers rather than verbal responses and encouraged participants to pause the discussion if they needed to immediately attend to anything. The researcher was also mindful that participants may have experienced a loss of agency at various times during their experiences, specifically to HCPs. As such, participants were encouraged to take the lead in discussions, sharing whatever themes were most salient to them, whether or not they reflected the questions asked. In this way, it was hoped that participants would feel a sense of control over the interview process and be more willing to share their experiences.

## Awareness of wider social and political context

As participants were initially recruited through the Australian Breastfeeding Association, MVR discussed with the principal investigator (JS) the fact that participants might have a bias in favour of breastfeeding or have some political position regarding government policy in relation to Covid control measures or vaccination. Given the non-generalisable nature of the study, it was thought that any such biases would be accepted as an intrinsic component of participants’ idiosyncratic world views, however, their explicit inclusion would only be warranted where they related to the research questions or provided further clarity on participants’ experiences. Whilst there is always the potential for political or philosophical differences between researchers and participants, MVR did not experience any need to ‘hijack’ the interview in order to present his own commentary on a participant’s experience or belief. The success of this may be attributed to a close adherence to the interview schedule on the researcher’s part and enabling participants to lead the discussion, with MVR taking a ‘back seat’ approach.

## The role of the research members as collaborators in knowledge production

Whilst this study formed part of the primary researcher’s honours thesis, its success can be attributed to the collaborative efforts of the primary researcher and principal investigator as well as the material support provided by other researchers attached to the wider research program. For example, both MVR and JS met regularly (at least once every two weeks) to discuss the progress of fieldwork and reflect on data collection. Meetings then intensified during the early stages of analysis in order to explore, discuss and debate emerging themes. In this endeavour, JS’ knowledge as a senior researcher, clinical psychologist and family therapist, as well as her lived experience as both a mother of older children and Victorian resident, were critical for revealing additional insights and research questions otherwise missed. Similarly, this study was nested within a wider international study presently exploring the impact of self-isolation and other Covid control measures on family functioning. As such, most of the interview schedule had already been developed prior to this study commencing and thus draws from the insights accumulated by the researchers engaged in those earlier studies.

## Potential for psychological harm

MVR was acutely sensitive to the possibility that discussing certain themes in the interview could potentially cause distress for participants. As such, MVR regularly checked-in with participants during the interviews and took the time to debrief participants at the end of each session. Whilst at times some participants did report feeling upset by the recollection of their experiences, when explicitly asked, all of the participants expressed the desire to continue with the interview.

# Appendix B: Deakin University Human Research Ethics Committee Approval

#
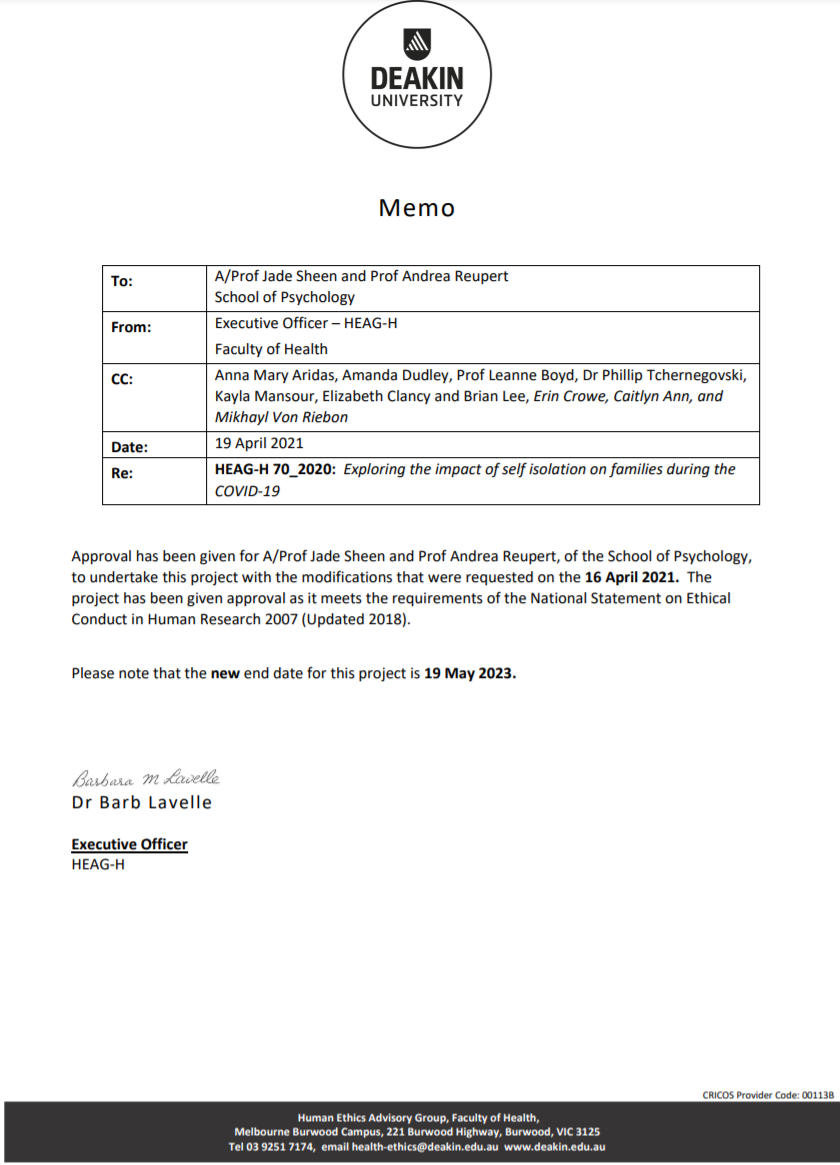


# Appendix C: Covid & Family Functioning - Interview Questions

## Initial Questions

To start, can you describe your family to me, who resides in your home?

Are you an expecting parent, and if so how far along is the pregnancy?

Is this your first pregnancy?

(If not already answered) how old are your children?

## Family Life

Have there been changes in family life since or due to the restrictions and social distancing?

Follow up: have you noticed specific changes in your:

Routine; such as getting up and going to bed? Meal times? When you work or study?

Daily habits; such as exercise, leisure time, what you eat

Family rituals; things you do as a whole family

Potential Probes: Tell me more about….? Can you give me an example of ….? What is the impact of this on you…? If no changes, why?

## Family Roles

Have any changes occurred in family roles since or due to the restrictions and social distancing?

By family roles, I mean who does the cooking, the cleaning, mowing the lawn and so on and whether this has changed?

What has been the impact of this on you?

And on your family (may need to go through different impact of different family members)

Potential Probes: Tell me more about….? Can you give me an example of ….? What is the impact of this on you…? If no changes, why?

## Experience as a Parent

Tell me about your experience as a new or expectant parent during the pandemic?

### Expectant Parent Prompts

Have there been impacts on things like appointments and preparing for the baby’s arrival? How have you managed this?

Can you tell me about the emotions and feelings you’ve experienced? How have you managed this?

Has the pandemic impacted the types of worries you have had during pregnancy? Tell me about these. How have you managed?

Have there been impacts on your mental health? Tell me about this. How have you managed this?

Have there been impacts on your connections with others? Have you felt more or less isolated? How have you managed this?

### New Parent Prompts

How did the pandemic impact your birthing experience/your baby’s arrival, if at all?

What was the impact of this on you? Your partner? Your other children?

How has the pandemic impacted life at home with your baby, if at all?

## Routines, parenting roles, boundaries, visitors

Has the pandemic impacted the types of worries you’ve had about your baby or yourself? Tell me about these. How have you managed?

Have there been impacts on your mental health? Tell me about this. How have you managed this?

Have there been impacts on your connections with others? Have you felt more or less isolated? How have you managed this?

Do you feel like you have received adequate support throughout your pre and post-natal experience? Tell me more about this. How have you managed this?

Has your experience as a new or expectant mother been different to what you expected or anticipated and if so in what way?

Has the pandemic impacted you and your family’s plans post maternity/parental leave?

## Breastfeeding/Lactation

What were your views regarding lactation (breastfeeding) prior to the delivery? Were you able to do this after the birth of your child?

Do you feel the pandemic influenced your decisions around breastfeeding. If yes, how so? (follow up, have you had any specific concerns regarding risk of COVID transmission during breastfeeding?)

What supports were you aware of in regards to breastfeeding?  E.g. familial support? Support through Health care services?

## For Parents with Older Children

Has the pandemic and restrictions impacted the way you are parenting your other children?

Discipline?

Shared parenting with partner?

The way you show affection?

Potential Probes: Tell me more about….? Can you give me an example of ….? What is the impact of this on you…? If no changes, why?

## Family Relationships

Have there been changes to family relationships and dynamics?

Prompt for relationship with partner?

Relationship with child/ren?

Relationship between partner and child/ren?

Sibling relationships?

Potential Probes: Tell me more about….? Can you give me an example of ….? What is the impact of this on you…? If no changes, why?

Has anything changed about how your family communicates or talks to each other?

Has anything changed about how your family support each other?

Have family boundaries changed, by which I mean the privacy family members have or the things that family members are allowed to do or not?

## Strengths

Have there been any advantages to having a baby during this period? If so, what are they?

What have been the family strengths that you and your family have drawn on throughout this period? What has helped you get through

What should parents of extremely young children and expecting mothers and fathers know about parenting during pandemics and large-scale disasters, such as COVID-19?

## Conclusion

Those are all the questions I have for you.

Do you have any additional comments or observations about family functioning during this period?

## End of Interview

# Appendix D: Themes, Subthemes and Exemplificative Quotations

| Superordinate theme | Subtheme | Exemplificative quotations |
| --- | --- | --- |
| T1: The ‘Control’ in Control Measure | Changes in Protocols | “My anxiety was a lot higher because things were ever changing and I guess I had no stability in what to expect and you’re already going into labour not knowing what to expect.” (Cordelia)“…you definitely felt the protocols had changed but it didn’t worry me one way or the other you know it was just, you noticed.” (Felicia)“…so we had to go for Covid tests beforehand which is hard with a baby because you don’t know when they’re going to come.” (Felicia)“…advice kept changing, even between different hospital staff as to what would be allowed at the time. Whether I would need a Covid test, whether I would have two people at the birth … or just my partner. Whether I would be allowed to have certain pain relief options…” (Georgia)“The midwife said, “oh well we’ve actually closed the water – the birthing pools because of Covid” …I was like, all right, so it’s impacting my whole pregnancy with all my appointments, now it’s impacting my preferred birth option.” (Irene) |
|  | Regaining Control | “I sought a mental health plan from my GP …just before I had the last IVF transfer and saw a psychologist …it got me through the most anxiety provoking stage, like that first trimester.” (Beatrice)“…because we went through private [husband] was able to make all of the scans which was …the toughest part for me because of my IVF history.” (Beatrice)“I don’t think that Covid itself had a huge impact on my labour and birth and I think that the main reason for that was my midwife and I knew her quite well and I think she made the whole process less stressful and she was very calming…” (Cordelia)“…what I chose to do was to …pay for a private obstetrician and go down that route and then go to a public hospital. …in terms of my experience, it was, it was really nice” (Delilah)“I tried really hard to be prepared, to prepare myself as much as possible, by trying to check up on what …the hospital restrictions- how they were changing week on week” (Georgia)“I wrote a specific breastfeeding plan and gave it to my midwife before I went into theatre. And I said, I want skin-to-skin as soon as possible, I want to try and breastfeed. And she goes, oh we’ll see what we can do. And I said, no, I’m – that’s – that’s what I want. I need to be able to do that.” (Irene) |
| T2: Care in the Time of Covid | Compared to Covid | “I feel like sometimes they forget that …there is a problem right in front of you that’s real – like Covid is real of course, but like …I feel like sometimes …I didn’t matter compared to Covid.” (Annie)“From the health care providers that I have proactively sought …I felt like those two examples went out of their way to make me feel supported. From the public side of things …I have not felt supported at all.” (Beatrice)“I’d spoken to a girlfriend who was a first-time mum, she was going public. And she said oh like I’ve hardly been there, they didn’t want us to come in and I thought oh okay. …that’s a bit weird” (Delilah)“While there was that fear on both parts of being a pregnant mum going into hospital and also for the hospital having, getting pregnant mums infected …there were other ways that you could feel supported …and I’m really glad I made that choice to do that” (Delilah)“…sometimes it felt like pregnant women …weren’t being prioritized …from the beginning it felt like the preparations for Covid meant that support opportunities were denied” (Georgia)“…I didn’t feel [the medical supports] were ideal, not for any medical professional’s doing but because of the lockdown situation, I didn’t feel as supported as I could have during pregnancy.” (Georgia)“…they had actually just started stockpiling furniture in the birthing suites with the hot tubs …So – because of Covid …they needed more room for extra beds.” (Irene) |
|  | At Arm’s Length | “Well the other thing that was hard during the pregnancy was reduced appointments, having to fight to get seen and not do Telehealth.” (Annie)“…even just logging into a Zoom meeting and getting the camera sorted and being able to hold a conversation with the camera whilst holding a baby is really difficult.” (Beatrice)“There’s things that you can’t really …assess via a phone or a computer and it’s a lot less personal, and particularly, things like breastfeeding is quite an emotional thing for a lot of people so I think face to face support provides a lot more for that.” (Cordelia)“…you don’t want to go to a doctor’s surgery …if you could avoid it, you don’t want to be anywhere like that.” (Delilah)“I did have a couple of telehealth appointments with the midwife through my obstetrician, but they were great …they gave you the option of telehealth if you wanted to or if you wanted to go in.” (Delilah)“[you’re] not necessarily as honest or you know forthcoming when you feel like you’re on a phone call and someone’s watching the clock.” (Felicia)“…breastfeeding classes over zoom …it’s more of a physical training experience in a way” (Georgia)“It was more difficult to ask questions just because it’s a very awkward format and …they weren’t as long as they would have been if they were in person.” (Georgia) |
|  | Masks and Midwives | “…you are pretty much dying and all you can see is these people with masks.” (Annie)“…I saw his whole face which was quite kind of shocking …he’s been treating me for 9 or 10 months and I’ve never really seen his face.” (Beatrice)“All of the nursing staff and doctors wore masks the entire time even in the …birthing ward. So that made it difficult just to communicate or even see a nurse’s expression which those nonverbal cues are really important to establishing effective communication.” (Beatrice)“everyone in the house had to check their temperature before they would come out… They had to have protective gear on and the mask and shield and that and then more as the labour progressed.” (Cordelia)“I think through the whole course of anti-natal appointments with everyone wearing masks, that’s a real barrier.” (Georgia)“I wasn’t wearing a mask but I was the only one beside from the baby not wearing a mask and that was a really weird de-humanizing experience …it felt really strange.” (Georgia)“Routine started with special care nurses coming in every other day with their face masks on and their shields on, sterilising their hands constantly.” (Irene) |
|  | Under Pressure | “I think I wasn’t ready to be discharged. I had the worst physical recovery of any of my pregnancies, like it took me like six weeks …I was not well.” (Annie)“…someone else had overhead the situation, someone more senior …who was able to look at it just with some common sense.” (Annie)“…sometimes it felt like everyone was very very rushed and I don’t know how that might have affected some of the support I’ve got in hospital.” (Georgia)“the nurse I had …was pretty anxious about seeing the community so it was definitely a stressful experience with the baby crying and getting measured…” (Felicia) |
| T3: Baby Space | It Takes A Village | “…having those little activities and little core groups and classes and whatever …they were also social things for me as well to chat to mums and connect” (Annie)“…the ability to meet other expectant parents was non-existent, we really didn’t have any mechanism at all.” (Beatrice)“I felt anxiety at or uncertainty over a whole range of topics that I think having that village around you would have helped alleviate so much quicker.” (Beatrice)“…everything was pulled back – classes, birthing classes, breastfeeding support, all the sort of clinics, pelvic floor clinic, all those sorts of things were all stopped.” (Cordelia)“…we did a group kind of physio class and …they would come to you to stop …people circulating” (Felicia)“…as a private patient they have a hotel program were you’re able to go if you’re feeling well, get transferred out of a maternity ward to a hotel ward, that was all shut down so that was a really big disappointment…” (Felicia)“…my parent’s group started quite a bit later than it would have if not for Covid so I was pretty desperate at that point for some sort of in-person support network...” (Georgia) |
|  | Together Alone | “…I guess being the third it wasn’t as bad but still it would have been nice” (Annie)“It took him longer to catch on that we were having a baby …Obviously he knew but …I feel like he had trouble engaging in those conversations earlier …and I wonder whether because it wasn’t as real for him …as it was for me.” (Annie)“I was very lucky I went through the private system, so I was able to …keep all those normal appointments and actually [husband] was able to attend I think all bar one of them due to the varying levels of lockdown.” (Beatrice)“…It was definitely a lot more separate than it had been with the previous pregnancies. …it was very much me on my own…” (Cordelia)“He was like “that’s not fair” …you know both for support for me and also for him to experience.” (Delilah)“…we’d had experiences of miscarriage, quite a few and there was quite a bit of anxiety about going for scans and not having [husband] there was a bit shit.” (Delilah)“I was checking the website to see and talking with my doctor to see if my husband was even allowed to stay” (Felicia)“…it was also just really sad that my partner [couldn’t] come to my appointments” (Georgia)“In normal times, I could have had him there …all day, just had a chat, hand him the baby …it was very isolating.” (Irene)“…that would have been nice to have him there when I heard that, because I had to wait to get home …instead of him hearing firsthand.” (Irene) |
|  | Our Little Bubble | “…in the end generally they hear you, but it’s taken away a bit of that mental space from just enjoying this little one.” (Annie)“…so visitors were limited …and in a way that was kind of good because we could just be safe in our own little bubble.” (Beatrice)“I just feel like we’ve not been able to share the happiness, like …it does definitely feel like there’s been a couple of barriers to prevent you from feeling all of those great feelings about being a new parent.” (Beatrice)“…after it got beyond …the first few weeks …I found it really difficult and I felt very angry...” (Cordelia)“We were able to spend those early weeks just at home absorbing our new baby.” (Cordelia)“It was just like so we could just all come back to each other it was nice.” (Delilah)“…the hospital was a lot calmer, like nobody else was having visitors, there was less people on the floor, less noise. I think the staff were more relaxed because they were just dealing with patients…” (Felicia)“So …they were the two …kind of feelings, of missing out and permission to just focus on you and the new baby.” (Felicia)“…it slowed the visitors to a drip feed which it might have been overwhelming otherwise.” (Georgia) |
